# The Impact of Rotavirus Vaccination on Discharges for Pediatric Gastroenteritis in Italy: An Eleven Year (2009–2019) Nationwide Analysis

**DOI:** 10.3390/vaccines11061037

**Published:** 2023-05-30

**Authors:** Claudia Isonne, Daniele Petrone, Martina Del Manso, Jessica Iera, Alessandra Caramia, Lorenzo Bandini, Giulia Fadda, Adriano Grossi, Valentina Baccolini, Claudio Costantino, Patrizio Pezzotti, Andrea Siddu, Fortunato D’Ancona

**Affiliations:** 1 Department of Infectious Diseases, Istituto Superiore di Sanità, 00162 Rome, Italy; 2 Department of Public Health and Infectious Diseases, Sapienza University of Rome, 00185 Rome, Italy; 3 Department of Statistics, Sapienza University of Rome, 00185 Rome, Italy; 4 Management and Health Laboratory, Institute of Management—Department EMbeDS, Sant’Anna School of Advanced Studies, 56127 Pisa, Italy; 5 Policlinico Riuniti Foggia Hospital, Hygiene Unit, Department of Medical and Surgical Sciences, University of Foggia, 71122 Foggia, Italy; 6 Department of Health Promotion Sciences, Maternal and Infant Care, Internal Medicine and Excellence Specialties “G. D’Alessandro”, University of Palermo, 90127 Palermo, Italy; 7 Ministry of Health, Directorate General Health Prevention, Communicable Diseases and International Prophylaxis, 00144 Rome, Italy

**Keywords:** vaccination, rotavirus, rotavirus vaccine, pediatric gastroenteritis, discharges

## Abstract

In Italy, despite the documented positive effects of rotavirus (RV) vaccination on reducing the burden of RV disease, an updated national assessment of its impact on clinical outcomes is still lacking. This study aims to analyze the implementation of RV vaccination in Italy, evaluating its impact on discharges for acute pediatric gastroenteritis (AGE). A retrospective analysis, including hospital discharge records and data on vaccination coverage for children aged 0–71 months from 2009 to 2019, was conducted. We examined trends in hospital discharge standardized incidence before and after vaccine introduction using a negative binomial mixture model with fixed effects to evaluate the impact of universal vaccination. The percentage of vaccination coverage increased over the years, from <5% between 2009 and 2013 to 26% in 2017, reaching 70% in 2019. The standardized incidence of discharges decreased over the period from 16.6/100,000 inhabitants in 2009–2013 to 9.9/100,000 inhabitants in 2018–2019. In this phase, about 15% of the estimated hospital discharges were avoided compared with those estimated in the first phase. The implementation of RV vaccination reduced AGE incidence discharges in children aged 0–71 months. Further efforts are needed to continue monitoring the vaccination effect over time and to increase vaccination coverage.

## 1. Introduction

Rotavirus (RV), a virus of the Reoviridae family, is the most common cause of severe dehydrating diarrhea in children under 5 years, with an associated high rate of morbidity and mortality [1,2]. It accounts globally for more than 25 million outpatient visits, whereas more than 2 million hospitalizations are attributable to RV infections each year [3]. The highest incidence rates are for children aged 6–23 months, and nearly every child develops RV infection once before the age of five [4]. Rotavirus is also one of the main causes of nosocomial gastroenteritis in pediatric patients, causing several hospital-acquired outbreaks [5]. While the incidence of RV disease in high-income and low-middle-income countries is similar, 80% of deaths occur in developing countries [6]. There are 10 RV antigenically organized groups (A–J), three of which cause disease in humans [5]. Transmission occurs primarily by the fecal-oral route, either directly from person to person or indirectly via contaminated fomites [7]. Over the years, despite efforts to reduce the burden of the disease, including improvements in water supply, hygiene, and sanitation, the negative impact of RV infections has remained high, indicating that the disease cannot be controlled exclusively with such measures [8].

Currently, there are two live oral RV vaccines licensed for global use. RV5 (RotaTeq—Merck and Co., Westpoint, PA, USA) is a pentavalent vaccine based on five bovine–human reassortant rotavirus strains (G1, G2, G3, G4, and P1A), and RV1 (Rotarix—GlaxoSmithKline, Rixensart, Belgium) is a monovalent vaccine based on a single human rotavirus strain (G1P). Although they differ in composition and schedule of administration, both can prevent severe RV gastroenteritis (AGE) caused by common RV genotypes, including G1–G4, G9, P [4], P [6], and P [8,9,10]. Specifically, vaccines’ effectiveness varies depending on the country’s mortality rate. In low-mortality countries, the vaccine has been found to prevent between 90% and 96% of severe rotavirus diarrhea cases in children who were followed up for a period of 2 years. On the other hand, in high-mortality countries, the vaccine has shown a lower but still substantial level of effectiveness, preventing between 35% and 54% of severe rotavirus diarrhea cases [11]. Since they became available, many countries have implemented national RV vaccination programs [9,12] with a consequent public health impact in reducing the burden of the RV disease, so much so that the World Health Organization (WHO) recommends the use of RV vaccination in all national immunization programs, particularly in countries with high diarrheal mortality rates among children [13]. Today, vaccination against RV represents the most effective strategy to counteract pediatric acute gastroenteritis (AGE). Its high effectiveness has reduced the incidence, hospitalizations, and economic burden of the disease [14,15,16]. Since 2017, RV vaccination has been introduced into routine pediatric immunization programs in several European countries [17]. Currently, 123 countries have introduced RV vaccines into national or subnational vaccine programs, while 16 countries are planning their introduction [18].

In Italy, the healthcare system is characterized by a Beveridge-like model with a high degree of decentralization and 21 different regional health systems [19]. The vaccination was adopted progressively in some of the 21 Italian regions and autonomous provinces (AAPPs) and was finally added to the Italian National Immunization Plan 2017–2021, with the objective of offering it to all newborns from 1 January 2018 [20,21]. According to the Italian Ministry of Health, RV vaccination coverage has improved in recent years without reaching the targets set in the plan [22]. The vaccination schedule is different depending on the vaccine, with RV5 requiring three doses and RV1 requiring two doses [23]. At the present time, voluntary vaccination is universally recommended for all children from the sixth week of life, and the vaccination course must be completed no later than 24 or 32 weeks of life, depending on the vaccine used [21,24]. Although the positive impact of vaccination coverage implementation over the years in some Italian regions has been highlighted [25,26,27,28], an updated national assessment of the vaccination impact on clinical outcomes is still lacking. This study aims to analyze hospital discharges for AGE in children ≤5 years old in Italy before and after the introduction of universal RV vaccination at the regional level in order to assess its impact over the years.

## 2. Materials and Methods

### 2.1. Study Population

For this study, we considered the resident children in Italy aged 0–71 months at the beginning of each year from 2009 to 2019 as the population under study. The years 2020 and 2021 were excluded as the pandemic could have affected the reliability of the data by not providing a picture comparable with the other years.

### 2.2. Study Design and Data Sources

We conducted a retrospective analysis using individual data from the Italian hospital information system from 2009 to 2019. This system routinely collects data on all hospital discharges, including both demographic and clinical information, primary diagnosis and up to five secondary diagnoses, primary and secondary diagnostic/therapeutic procedures, type of discharge, and hospital length of stay. The information comes from public and private hospitals and covers 100% of hospital discharges in Italy. Diagnoses and procedures are coded using the ‘‘International Classification of Diseases, 9th Revision, Clinical Modification” (ICD-9-CM) [29]. We excluded patients who were admitted to hospital within 14 days of their previous discharges.

As for AGE hospital discharges, they were included if at least one of the following ICD-9-CM codes were used in any diagnosis position: 00861 (rotavirus enteritis), 0090 (colitis, enteritis and gastroenteritis of infectious origin), 0091 (diarrhea and gastroenteritis of presumed infectious origin), 0092 (diarrhea of infectious origin), 0093 (diarrhea of presumed infectious origin), 5589 (elsewhere and of unspecified gastroenteritis and colitis of non-infectious origin), 5645 (functional diarrhea), and 78791 (diarrhea).

**Figure 1:**
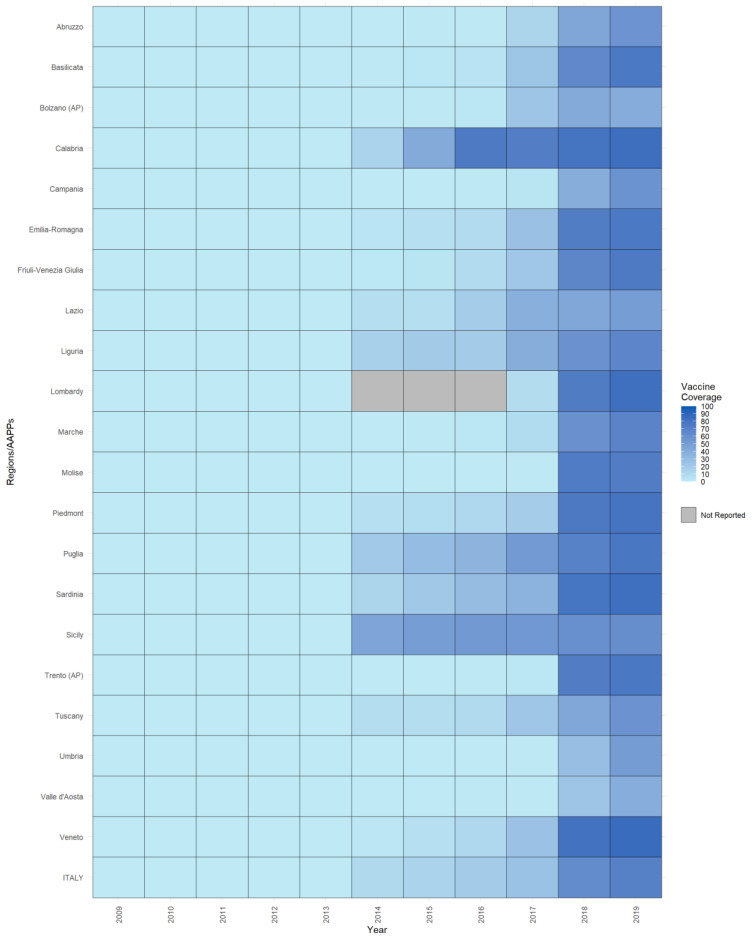
Regional RV vaccine coverage by year, 2009–2019. The increasing intensity of color represents the increase in regional vaccination coverage over time.

Data about RV vaccination for the same period was obtained from the Italian Ministry of Health (MoH), which routinely receives data from the 19 regions and the two autonomous provinces (AAPPs) and collects yearly anonymized and aggregated data on administered vaccines (numerator) and the target resident population (denominator) of the same age (i.e., per birth cohort) through the local health authorities, according to their organization models. Data for infants are collected at 24 months. Since 2019, the Ministry of Health has also collected data for rotavirus vaccination at 12 months of age [30]. Based on this data, 14 regions/AAPPs achieved at least 50% vaccine coverage in the 2018 cohort, and the number increased to 19 regions in 2019 (Figure 1). In order to standardize the national and regional incidence of hospital discharge rates by age, we used the 2020 Italian resident population, obtained from the National Institute of Statistics (ISTAT). The population data were extracted from the data warehouse (https://demo.istat.it/app/?i=POS&l=it; accessed on 11 December 2022).

### 2.3. Statistical Analysis

Analysis was restricted to cases 0–71 months of age at the time of AGE hospital discharges and between 2009 and 2019. The main characteristics of AGE cases and the frequency of the outcomes of interest were described using counts and percentages. Time series analysis techniques were used to evaluate the overall trend and the seasonality of AGE, as well as the effect of the vaccination introduction. Weekly AGE hospital discharges were considered. To analyze the trend in AGE hospital discharge, we used a Negative Binomial Model with fixed effects where we estimated the age-standardized incidence adjusted by date (progressive number of weeks from 2009 to 2019), vaccine coverage phase (first phase: 2009–2013, second phase: 2014–2017, and third phase: 2018–2019), annual seasonal components (estimated by sin (2πdate/52) and cos (2πdate/52)), and the interaction term among coverage phase and date and among coverage phase and seasonal components. We calculated the number of avoided hospital discharges as the difference between the number of observed hospital discharges and the estimated number of them before the introduction of the vaccination (i.e., the first phase of the vaccine coverage, 2009–2013). We carried out a supplementary analysis where we considered only hospital discharges due to rotavirus enteritis (ICD-9-CM code: 00861). All the analyses and the figures were carried out with RStudio 2023.03.0 under R 4.2.2.

## 3. Results

### 3.1. Vaccination Implementation

In Italy, rotavirus vaccination was introduced at different times and offered to children in different ways. Children could access vaccination with a copayment from 2010 in the Piedmont Region, from 2013 in Friuli-Venezia Giulia and in the AP of Bolzano, and from 2014 in Veneto and Puglia. In the same years, in Piedmont, Friuli-Venezia Giulia, and the Veneto Region, at-risk children could be vaccinated for free. In 2015 and 2017, Lombardy and Emilia-Romagna, respectively, offered free vaccinations for at-risk subgroups. In Emilia-Romagna, since 2017, vaccination has been accessible for free but not actively implemented. Active and universal vaccination was first introduced in Sicily in 2014, followed by Calabria and Puglia in 2015, and Sardinia in 2016. Between 2017 and 2018, the other regions/AAPPs progressively offered the vaccination universally and actively (Figure S1 of the Supplementary Material). All the regions and AAPPs reached at least 50% of RV vaccine coverage, except one region and one AP (Figure 1).

### 3.2. Hospital Discharges

From 2009 to 2019, we included in the analysis 276,981 AGE hospital discharges from all the Italian regions/AAPPs. No difference in sex distribution was observed. They were more frequent in young children (55.8% of those admitted were aged 0–11 or 12–23 months), and their number decreased as age increased. The most frequent ICD-9-CM code was 0091 (diarrhea and gastroenteritis of presumed infectious origin; *n* = 89,080; 32.2%); the number of AGE hospital discharges with an RV diagnosis (ICD-9-CM: 00861; rotavirus enteritis) was 82,590, about 30% of the total (Table 1).

In the first part of the study period, when RV vaccination was implemented only in a few regions (i.e., from 2009 to 2015), the average yearly national standardized incidence was over 14 discharges per 100,000 inhabitants; in the last four years of the study period, it became lower, with a peak of 13 discharges per 100,000 inhabitants in 2017 (Table 2).

**Figure 2:**
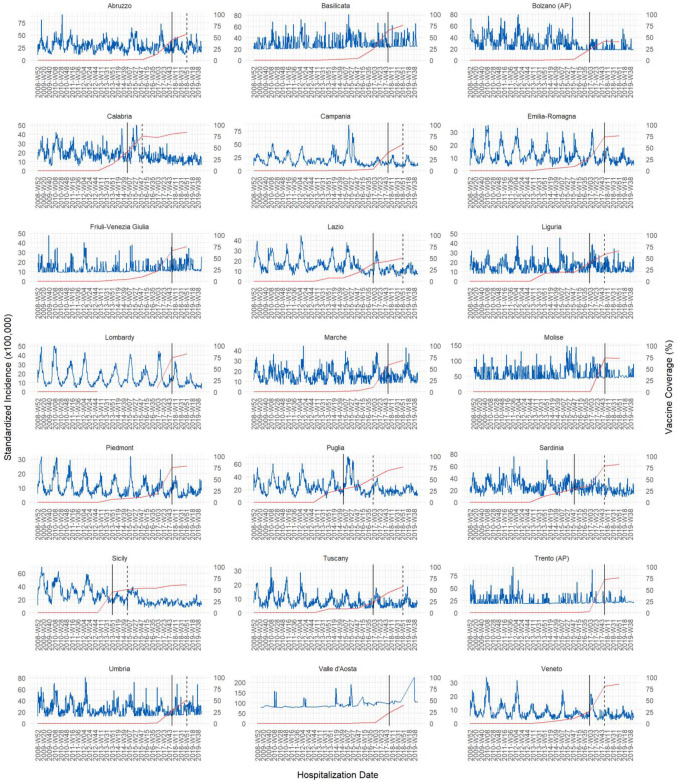
Monthly standardized incidence trend (×100,000 persons) and RV vaccination coverage by Italian region and autonomous province, from 2009 to 2019. Blue line: standardized incidence trend of discharges for all the 21 regions/AAPPs. Red line: yearly regional RV vaccine coverage. Vertical dotted black line: first year when the region/AP reached at least 50% vaccine coverage. Vertical continuous black line: year of RV vaccination introduction.

In each region/AP we observed a clear seasonal effect, with the highest standardized incidences of AGE hospital discharges usually in the spring/winter period (Figure 2). The standardized incidence trend of discharges, for all the 21 regions and AAPPs, was declining (blue line in Figure 2) during the study period, in particular after the introduction of RV vaccination (right to vertical continuous black line in Figure 2). The vertical dotted black line represents the first year when the region/AP reached at least 50% vaccine coverage; the red line indicates the yearly regional RV vaccine coverage.

**Figure 3:**
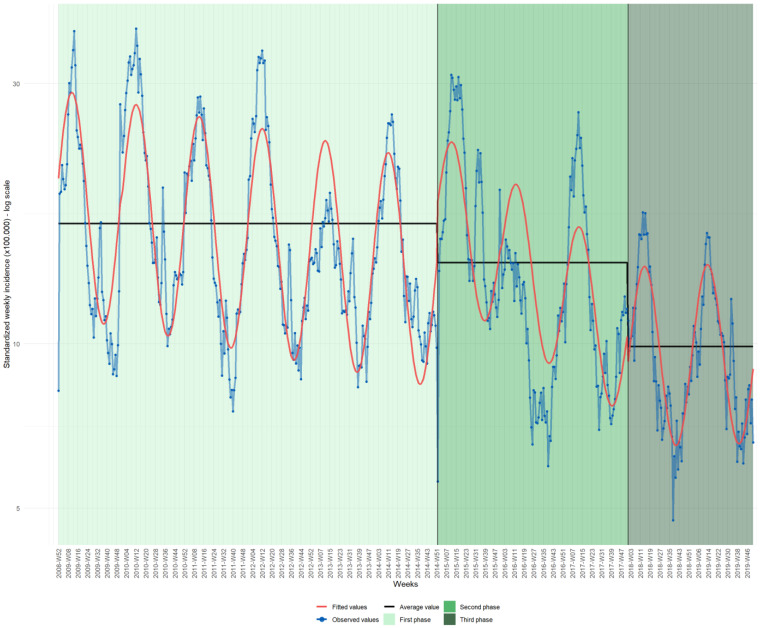
Weekly incidence rates of AGE hospital discharges in Italy; hospital discharge record system, 2009–2019; 0–71 months.

During the three phases examined, the national average incidence of hospital discharges decreased (Figure 3), from 16.6 discharges × 100,000 inhabitants in the first phase to 14.1 discharges × 100,000 inhabitants in the second phase, and finally to 9.9 × 100,000 inhabitants in the third phase. Figure 3 shows peaks during the winter-spring period.

The negative binomial model applied to all the hospital discharges shows that in the second phase there was no evident reduction in the actual rates compared with those forecast using phase one data, except for the year 2016, when 20% of avoided hospital discharges were observed compared with those forecast (Table 3 and Figure S2 of the Supplementary Material). For the years 2015 and 2017, respectively, 34.2% and 5.2% over the forecasted rate of hospital discharges were observed. During the third phase, on average, 14.8% of avoided hospital discharges per year were observed.

The supplementary analysis carried out only on rotavirus hospital discharges shows a greater percentage of avoided hospital discharges in 2016, 2018, and 2019 (Table S1 and Figure S3 of the Supplementary Material).

## 4. Discussion

Since RV vaccination was implemented in Italy, hospital discharges for AGE have shown a decreasing trend over the years, confirming the remarkable positive impact of RV vaccination programs. Specifically, the results of our study suggest a relationship between the reduction of AGE hospital discharges and the increase in RV vaccination coverage at the national level, as observed in previous regional experiences [25,26]. In Italy, before the introduction of universal RV vaccination in their regional immunization plans in 2017, the regions/AAPPs took different approaches to providing access to RV vaccination, following different regional policies [28]. Sicily, for example, was the first region to offer universal RV vaccination in 2013, followed by Calabria in 2015; Puglia introduced RV vaccination for all newborns at a discounted co-payment, whereas other regions offered free vaccination to defined categories of infants [28]. These regional differences could explain the heterogeneous effect of RV vaccination in reducing the AGE hospital discharges that were observed in our study before the introduction of universal RV vaccination. The significant public health benefit of RV vaccination observed in our results was also previously reported across European countries, in which its positive impact on reducing the burden of healthcare utilization was observed in terms of hospitalizations and also in terms of nosocomial RV infections and considering outpatient visits [31,32]. Although the use of RV vaccines remains suboptimal, some progress has been made, so much so that, to date, almost half of European countries have fully funded universal mass vaccination RV programs [33]. Similarly, these substantial benefits have also been observed in the United States and Australia, where, in line with our findings, a notable reduction in the number of hospitalizations due to all-cause diarrheal disease has been documented in the postvaccine era [34,35]. Notwithstanding, the implementation of vaccination in countries around the world remains heterogeneous, and there are some aspects that need to be addressed to counteract the reluctance that some health authorities still show in promoting and implementing vaccination programs against RV [33]. It is essential to continue efforts to raise awareness among stakeholders about the benefits of RV vaccination and address any barriers or challenges that may be hindering its uptake in order to enhance protection against this common and preventable viral infection [33].

As regards hospital discharges for AGE, we found that, according to the RV epidemiology [36,37], they mostly occurred in children aged ≤ 24 months. Based on our findings, a significant percentage of prevented cases were observed in the third phase (2018–2019) of the study. These results highlight the importance of providing early protection against RV and implementing targeted and effective strategies for promoting newborn vaccination during the first year of life [23]. It is important to consider that RV vaccination is administered within the first year of life, and long-term catch-up schedules for children based on age whose vaccinations have been delayed are not available, as in the case of other vaccinations [21]. Vaccine administration according to the timing of the vaccination schedule also induces herd immunity, which has been observed following the regular administration of rotavirus vaccines [7]. This phenomenon occurs when the transmission of RV decreases within a community, resulting in indirect protection for individuals who have not been vaccinated [7]. Evidence collected after the implementation of RV vaccines reveals substantial decreases in RV disease among individuals who were either too old or too young to receive the vaccine [38]. In this context, it is crucial to consider organizational aspects that could influence vaccination uptake. Recent evidence showed that the difficulty in accessing routine vaccination services that occurred during the COVID-19 pandemic led to a worldwide reduction in vaccination coverage, leaving millions of children under-vaccinated or unvaccinated against preventable diseases [39]. Thus, facilitating access to health services appears to be essential to counteract the delay or skip of the vaccinations [40]. Alongside this, in order to achieve and maintain high vaccination coverage, other behavioral aspects that may influence vaccination acceptance must be taken into account. For example, evidence showed that, despite a significant level of parents’ worries and distress about AGE hospitalization, most of them were unwilling to vaccinate their children [41]. Among the reported reasons of interest is the fear of side effects such as intussusception, which has been observed in some cases following RV vaccination but whose correlation remains unclear [42,43]. In this regard, extended monitoring and surveillance indicated that RV vaccination seemed not to increase the occurrence of intussusception. By contrast, the achievement of early and widespread rotavirus vaccine coverage could result in a reduction in intussusception cases during the first year of life [33]. Thus, it is critical to increase the knowledge among parents on RV disease and on the availability of effective vaccines, and the benefits of the vaccination should be clearly explained and emphasized [41,44]. Health literacy could have a role in directing people towards vaccination behaviors, increasing their awareness when choosing to vaccinate their children [45]. At the same time, future research should focus on investigating barriers to vaccination uptake among parents in order to counteract vaccine hesitancy that has contributed to the recent increase in vaccine-preventable disease outbreaks registered worldwide over recent years [46,47]. In this context, there is a need to focus on the training of health professionals who could play a key role in influencing parents’ vaccine acceptability through effective and targeted communication strategies [48]. Pediatricians are often the main source of health information for parents, and the low percentages reported among those recommending vaccination highlight a worrying scenario [48]. Certainly, a low risk perception in terms of the severity of clinical forms and mortality of RV disease could be responsible for the lack of interest among physicians, especially in countries where the burden of the disease is considered lower [37]. Notwithstanding, since the negative impact of the disease remains higher globally, health policies should also focus on raising awareness among healthcare professionals to increase vaccine acceptability in the population [48]. These efforts become even more essential considering the downstream impacts of RV vaccination [33]. In fact, a link between the reduction in some autoimmune diseases triggered by RV infection and RV vaccination was observed in some studies. Nevertheless, research into the expanded benefits of RV vaccines on various clinical aspects is an ongoing and active area of study that needs to be increased and reinforced [33]. Lastly, it should be noted that our results could also have health economic implications: the reduction of AGE hospitalizations observed could represent a proxy for a large health-care cost savings attributable to RV vaccination, which was already found to be a cost-effective strategy from a health payer’s and societal perspective [28]. Previous economic analyses have shown that, in the absence of vaccination, RVGE would lead to increased healthcare costs and reduced quality of life in patients [49,50]. In Italy, according to the Italian Ministry of Health, it was estimated that the total direct costs of RV disease in the absence of vaccination (calculated on a population of children aged 5 and under) amount to EUR 31,471,642 per year. By adding indirect costs, the expenditure rises to EUR 143,908,762 per year [21]. The implementation of vaccination coverage thus seems to be a key factor also from the perspective of containing health care spending, as our National Health Service, which provides universal coverage largely free of charge at the point of service, has limited resources [51].

This study has several strengths and limitations. The main strength is the ability to provide evidence on the impact of RV vaccination over time, with available data on ten years of vaccination coverage and hospital discharges. Furthermore, since we used data from national registries, the generalizability of the results and the evaluation of the vaccine impact across different settings and populations were possible. By contrast, the main limitation of our study is related to the accuracy of the results of both the main and supplementary analyses. In fact, in the first case, the selection of all-cause acute gastroenteritis and not only those with laboratory-confirmed RV disease could have led to an overestimation of the RV diagnosis, even though this bias can be considered constant over time. On the other hand, given that probably not all the gastroenteritis was tested for RV, the inclusion of codes only related to RV gastroenteritis in the supplementary analysis could underestimate the results. However, we considered the two possibilities, demonstrating a decreasing trend over the years in both analyses. Another limitation could be due to the fact that, despite gastroenteritis being the main clinical manifestation of RV infection, we did not consider other atypical symptoms with which the disease might present. Lastly, we noted an anomalous trend for the years 2015 and 2016. This finding is probably due to inaccuracies or other issues related to the completion of hospital discharge records. However, to the best of our knowledge, this is the first study to provide an updated analysis of the ten year impact of RV vaccination in the pediatric Italian population, evaluating its effect over time throughout the country and using reliable and comparable data from national registries. Further studies are needed to assess the global burden of RV vaccination considering additional aspects such as the economic impact on the healthcare system. Furthermore, given the negative effect of the COVID-19 pandemic on pediatric vaccination rates, future research that considers the pandemic period could be useful to evaluate its consequences on RV vaccination coverage.

## 5. Conclusions

In this study, substantial reductions in AGE discharge rates, which followed the progressive RV vaccine introduction in the Italian regions, were observed. The reduction in the risk of hospital discharges followed a gradient as vaccination coverage increased, with regions/AAPPs with higher vaccination coverage rates showing lower discharge rates. Further efforts are needed to maintain high vaccination coverage, thus reducing the negative impact of rotavirus disease.

## Figures and Tables

**Table 1:** Characteristics of AGE discharges included in the analysis (*n* = 276,981), Italy, 2009–2019.

	Frequency	Percentage
**Sex**		
Female	125,204	45.2
Male	151,777	54.8
**Age in months**		
0–11	72,126	26.0
12–23	82,438	29.8
24–35	51,834	18.7
36–47	32,283	11.7
48–59	21,802	7.9
60–71	16,498	6.0
**ICD-9-CM codes**		
00861	82,590	29.8
0090	33,161	12.0
0091	89,080	32.2
0092	4810	1.7
0093	7449	2.7
5589	46,586	16.8
5645	351	0.1
78791	12,954	4.7

00861: rotavirus enteritis; 0090: colitis, enteritis e gastroenteritis of infectious origin; 0091: diarrhea and gastroenteritis of presumed infectious origin; 0092: diarrhea of infectious origin; 0093: diarrhea of presumed infectious origin; 5589: elsewhere and of unspecified gastroenteritis and colitis of non-infectious origin; 5645: functional diarrhea; 78791: diarrhea. ICD-9-CM: International Classification of Diseases, 9th Revision, Clinical Modification. AGE: Acute Gastroenteritis.

**Table 2:** Raw and standardized incidence of AGE discharges in Italy by year, from 2009 to 2019.

Year	Raw Incidence (×100,000 Persons)	Standardized Incidence (×100,000 Persons)
2009	17.8	17.2
2010	20.7	20.1
2011	16.7	16.2
2012	18.1	17.7
2013	13.6	13.3
2014	15.4	15.2
2015	18.7	18.6
2016	10.5	10.5
2017	13.2	13.2
2018	9.9	9.8
2019	10.0	9.9
AGE: Acute Gastroenteritis		

**Table 3:** Forecasted rates, actual rates, and avoided hospital discharges of AGE in Italy, 2015–2019.

Year	Forecasted Rates	Actual Rates	Difference between Rates	% of Avoided Discharges *
2015	13.9	18.6	−4.7	−34.2
2016	13.2	10.5	2.7	20.4
2017	12.5	13.2	−0.7	−5.2
2018	11.9	9.8	2.1	17.3
2019	11.3	9.9	1.4	12.3

* Obtained as difference × 100/forecasted rates; AGE: Acute Gastroenteritis.

## Data Availability

Not applicable.

## Supplementary Materials

The following supporting information can be downloaded at: https://www.mdpi.com/article/10.3390/vaccines11061037/s1, Figure S1: Year of introduction and type of offering by region/AP of rotavirus vaccination in Italy, 2009–2019; Figure S2: Observed and forecasted rates using the Negative Binomial Model with fixed effects on weekly rates of AGE hospital discharges in Italy, 2009–2019; 0–71 months; Figure S3: Observed and forecasted rates using the Negative Binomial Model with fixed effects on weekly rates of RV hospital discharges in Italy (ICD-9-CM code: 00861), 2009–2019; 0–71 months; Table S1: Forecasted rates, actual rates, and avoided hospital discharges of RV hospital discharges in Italy (ICD-9-CM code: 00861), 2015–2019.
