# Supplementary material for: The Impact of Rotavirus Vaccination on Discharges for Pediatric Gastroenteritis in Italy: An Eleven Year (2009–2019) Nationwide Analysis
Source: Vaccines (Basel). 2023 May 30;11(6):1037. doi: 10.3390/vaccines11061037 (PMC10300783; doi:10.3390/vaccines11061037)
Supplement: Supplementary file 1 [file vaccines-11-01037-s001.zip › vaccines-2373748-supplementary.pdf]

Supplementary Material

Figure S1 – Year of introduction and type of offering by Region/AP of rotavirus vaccination in Italy, 2009-2019.

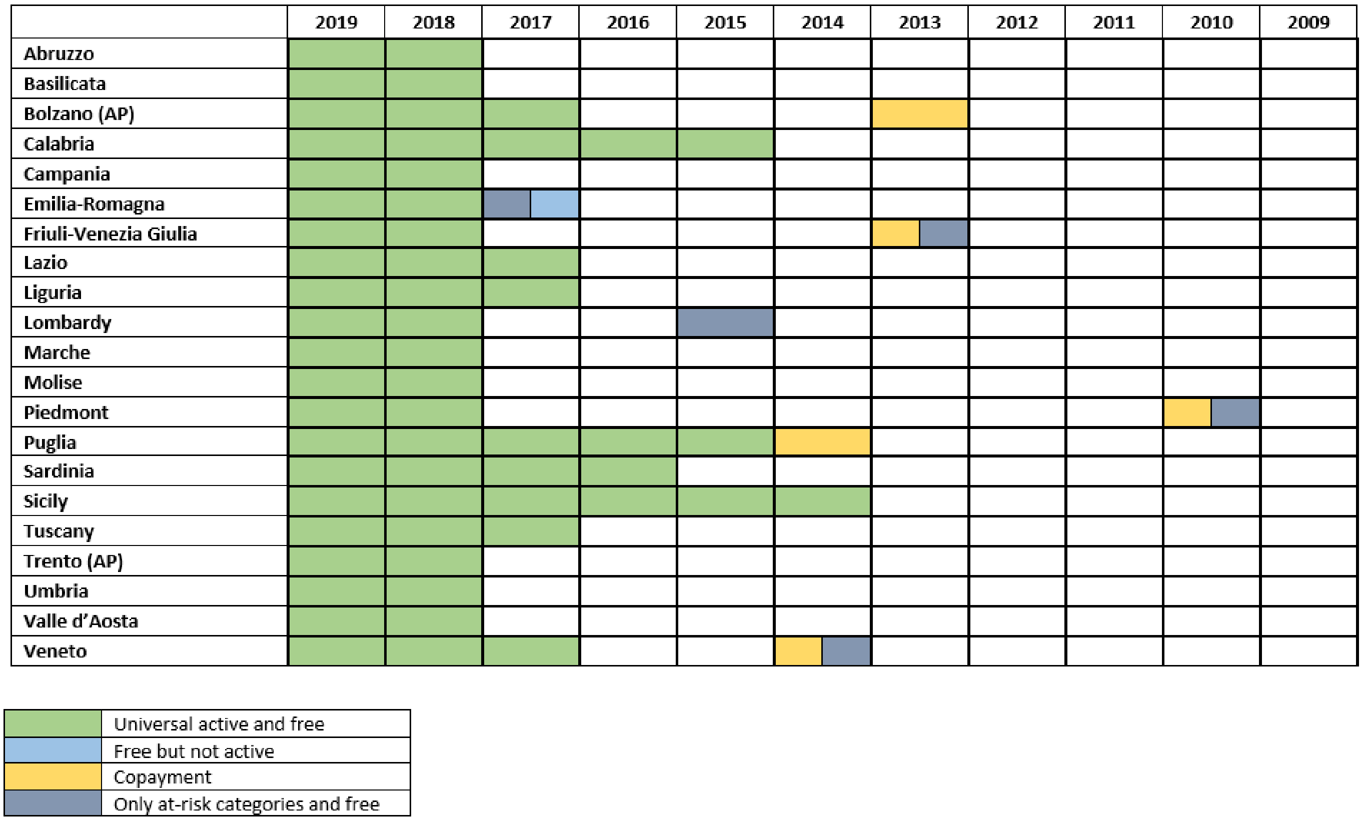

Figure S2 – Observed and forecasted rates using Negative Binomial Model with fixed effects on weekly rates of AGE hospital discharges in Italy, 2009-2019; 0-71 months.

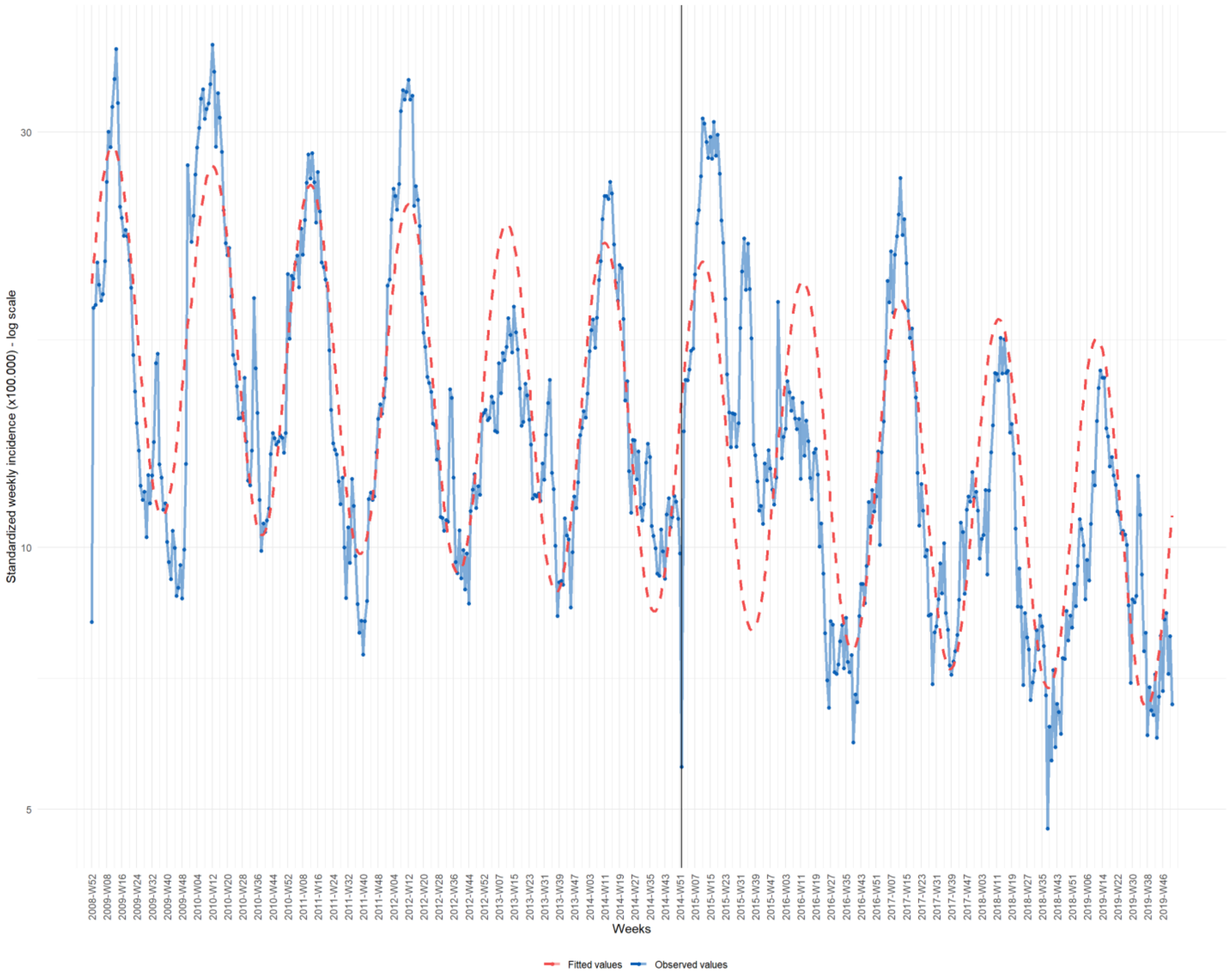

Figure S3 – Observed and forecasted rates using Negative Binomial Model with fixed effects on weekly rates of RV hospital discharges in Italy (ICD-9-CM code: 00861), 2009-2019; 0-71 months.

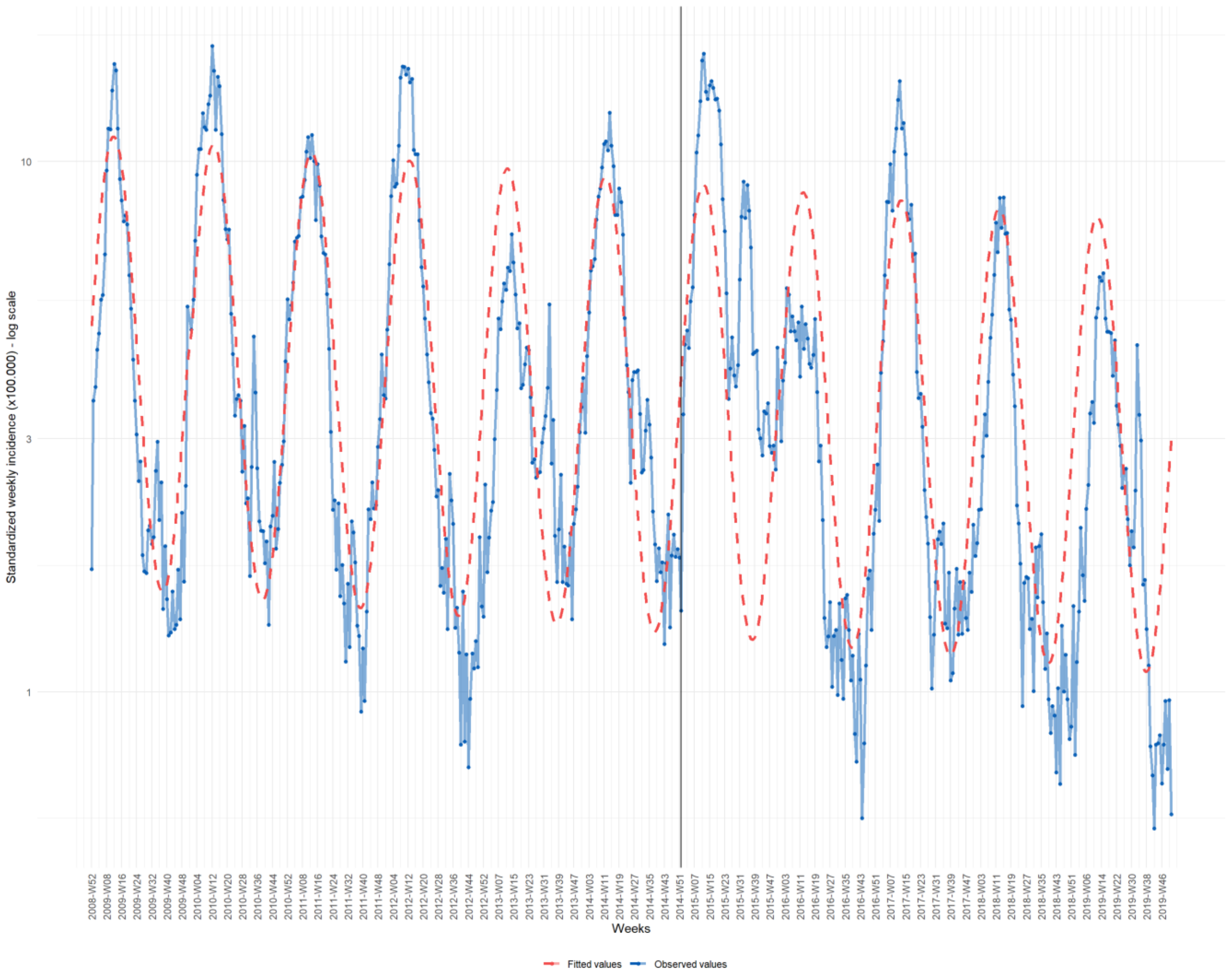

Table S1 – Forecasted rates, actual raters and avoided hospital discharges of RV hospital discharges in Italy (ICD-9-CM code: 00861), 2015-2019.

Year	Forecasted rates	Actual rates	Difference between rates	% of avoided discharges*
2015	4.2	7.1	-2.9	-67.7
2016	4.1	2.6	1.5	35.8
2017	4.0	4.4	-0.5	-12.2
2018	3.8	2.8	1.0	26.2
2019	3.7	2.6	1.1	31.0

* obtained as Difference x100/Forecasted rates; RV:Rotavirus
